# Are Older Adults without a Healthy Diet Less Physically Active and More Sedentary?

**DOI:** 10.3390/nu11051119

**Published:** 2019-05-20

**Authors:** Ming-Chun Hsueh, Ru Rutherford, Yi-Hsuan Huang, Hung-Yu Chang Chien, Chia-Hui Chang, Jong-Hwan Park, Yung Liao

**Affiliations:** 1 Department of Physical Education, National Taiwan Normal University, 162, Heping East Road Section 1, Taipei 106, Taiwan; boxeo@ntnu.edu.tw; 2 Institute of Sports Pedagogy, University of Taipei, No. 101, Sec. 2, Jhongcheng Rd., Shilin Dist., Taipei 11153, Taiwan; 3 Department of Health Promotion and Health Education, National Taiwan Normal University, 162, Heping East Road Section 1, Taipei 106, Taiwan; bluenstop@gmail.com (R.R.); vincent6403@gmail.com (Y.-H.H.); eric840617@gmail.com (H.-Y.C.C.); aj820927@gmail.com (C.-H.C.); liaoyung@ntnu.edu.tw (Y.L.); 4 Health Convergence Medicine Research Group, Biomedical Research Institute, Pusan National University Hospital, 179, Gudeok-Ro, Seo-Gu, Busan 49241, Korea

**Keywords:** accelerometer, questionnaire, physical activity, sedentary bouts, sedentary break, elderly

## Abstract

Few studies on older populations consider several energy balance-related behaviors together. This cross-sectional study compared subjectively and objectively measured physical activity (PA) and sedentary behavior (SB) patterns between older adults with and without a healthy diet. We recruited 127 community-dwelling older Taiwanese adults (69.9 ± 5.0 years); data were collected during April and September 2018. Objectively measured total PA, moderate-to-vigorous PA, light PA, step count, total sedentary time, duration of sedentary bouts, number of sedentary bouts, and number of sedentary breaks were assessed using activity monitors. Subjectively measured PA and SB were measured using the International Physical Activity Questionnaire and Sedentary Behavior Questionnaire for Older Adults. Chi-square tests and independent sample t-tests were performed. For subjective measures, older adults without a healthy diet spent significantly less total leisure time on PA and more leisure sitting time than those with a healthy diet. For objective measures, older adults without a healthy diet spent less time on light PA and had a higher total sedentary time, duration of sedentary bouts, times of sedentary bouts, and times of sedentary breaks than those with a healthy diet. Regardless of the use of objective or subjective measurements, older adults without a healthy diet engaged in a more inactive and sedentary lifestyle. These findings have implications for health promotion practitioners in designing tailored interventions.

## 1. Introduction

Energy balance-related behaviors (EBRBs) are behaviors that may influence the energy balance and include dietary behavior, sedentary behavior (SB), and physical activity [1,2]. Healthy dietary behavior, physical activity (PA), and reduced sedentary time decrease the risks of mortality, non-communicable diseases, and geriatric syndrome in older adults [3,4,5]. Despite the fact that dietary behavior, physical activity, and sedentary behavior play significant roles in older adults’ health, only a few studies on the elderly population have considered these three EBRBs together [6]. In particular, for older adults, it is important to further compare physical activity and sedentary behavior patterns of those with and without a healthy diet, because physical activity and sedentary behavior may play important roles in influencing functional ability [7,8]. This information is critical for designers of behavioral change interventions and health-promotion practitioners in designing tailored interventions for older adults without a healthy diet.

However, there is a major concern regarding the measurement methods in the existing studies [9,10,11]. Few studies have examined physical activity and sedentary behavior patterns using both subjective and objective measures [9]. Using objective measures such as accelerometers can help to precisely measure activity intensity (sedentary, light, moderate, and vigorous), activity patterns (long sedentary bouts and break times), and step counts [10]. Despite the limitation of recall methods, using subjective measures can reflect the awareness of participants’ physical activity and sedentary behaviors and provide information on the types, frequency, and duration of PA, which are all important health indicators [11]. Therefore, we used both objective and subjective measures to collect data on older adults’ awareness of and actual physically active and sedentary lifestyles. In addition, although several studies examined the association between physical activity and dietary behavior [12,13], few compared the sedentary behavior patterns of older adults with and without a healthy dietary behavior [14]. We hypothesized that older adults without a healthy diet may engage in less physical activity and more sedentary time than those with a healthy diet. To fill these research gaps, this study aimed to compare the patterns of physical activity and sedentary behavior between older adults with and without a healthy diet using objective and subjective measures.

## 2. Materials and Methods

### 2.1. Participants

The present cross-sectional study was conducted in Taipei city, Taiwan, during April and September 2018. The inclusion criteria of this study were being community-dwelling older adults and aged 60 years or older. Those who were unable to walk independently (were using any walking assistance devices) were excluded from the present study [15]. The participants were recruited through local advertisements and announcement in the community. The participants contacted the study recruiters if they were interested in participation. In total, 170 men and women aged over 60 years were enrolled. First, every participant completed an informed consent form and questionnaire about socio-demographic information, healthy behavior, health condition, physical activity, and sedentary behavior. Furthermore, the participants wore an accelerometer on their waist for seven consecutive days. A convenience-store gift certificate (worth 7 USD) was given after they completed the questionnaire and wore the accelerometer for the purposes of this study. Incomplete questionnaire and accelerometer data were excluded (*n* = 43). For the data analysis, 127 participants were included in the study.

### 2.2. Research Ethics

Our study was conducted following the Declaration of Helsinki of 1975 and its subsequent revisions. Before participation in the study, each participant provided written informed consent. We also obtained ethical approval from the Research Ethics Committee of the University (REC number: 201711HM003).

### 2.3. Subjectively Measured Physical Activity and Sedentary Behavior

#### 2.3.1. Leisure-Time Physical Activity

We used the Taiwanese version of the International Physical Activity Questionnaire-Long Version (IPAQ-LV) to measure leisure-time physical activity. The IPAQ-LV had acceptable validity (Intraclass Correlation Coefficient [ICC] = 0.79) [16]. We collected data on the time spent on different types of physical activity including physical activities of vigorous and moderate intensities and walking for the previous seven days.

#### 2.3.2. Leisure-Time Sedentary Behavior

Sedentary behavior was assessed using the 10-item Sedentary Behavior Questionnaire for Older Adults, which was validated in previous studies [17]. The questionnaire covered various types of sedentary behavior including TV viewing, computer using, reading, socializing, sitting in an automobile, eating, working (or volunteering), napping, hobbies (e.g., chess), and other sedentary behaviors (e.g., craft or class). Each item demonstrated reasonable test–retest reliability and validity and assessed the overall sedentary time. The participants were asked to recall how much time they spent on each sedentary behavior and how many days they performed these behaviors in the previous seven days. In accordance with the measures of leisure-time physical activity, three items (eating, napping, and working time) were excluded because eating and working are not leisure-time sedentary behavior, and nap is not a waking behavior [17]. The remaining seven items were summed as the variable total time spent on leisure-time sedentary behavior.

### 2.4. Objectively Measured Physical Activity and Sedentary Behavior

In this study, we used ActiGraph, which is an activity monitor (wGT3X-BT) that counts the steps and measures the time spent on total, light, moderate-to-vigorous physical activity, and sitting, as well as sedentary patterns including the duration of a sedentary bout, times of sedentary bouts, and sedentary breaks. We also used the ActiLife software (version 6.0, Pensacola, FL, USA) to analyze the accelerometer data. Triaxial accelerometer models have demonstrated high intra-monitor reliability and have been validated with acceptable criteria [18]. The accelerometer recorded data for seven days (five weekdays and two weekend days). However, sleeping time was not calculated as sedentary time but was classified as unfitted time. Non-wear time was defined as 60 min or more of continuous unbroken 0 counts with a tolerance of up to 2 min of limited movement. Participants with at least three valid days of accelerometer data and at least one weekend day were included in this study. A period of 600 min (10 h) or more of monitor wear time was categorized as a valid day.

Accelerometer counts can distinguish sedentary behavior from physical activity. Here, ≤99 counts/min was regarded as sedentary time, and ≥100 counts/minute as physical activity. For physical activity, light-intensity physical activity (LPA) was defined as PA between 100 and 2019 counts/min, and moderate-to-vigorous-intensity physical activity (MVPA) as greater than or equal to 2020 counts/min [19]. The MVPA bouts as periods of MVPA lasting at least 10 consecutive minutes with a one-minute allowance below the MVPA threshold were included in the analysis [18]. In this study, LPA, MVPA, total PA (LPA + MVPA), and daily step counts were included as exposure variables. Following previous studies [20,21], total sedentary time, number and duration of ≥30 min sedentary bouts were calculated for the analysis. The drop time of a sedentary bout was set at two minutes when the data were analyzed.

### 2.5. Healthy Dietary Behavior

Participants were asked to report whether they had a healthy dietary behavior based on the requirements of six key nutrient groups daily, according to the current Taiwanese dietary guidelines [22]. The six nutrient groups include the following: (1) miscellaneous, (2) milk and dairy product, (3) vegetables, (4) fruits, (5) soybean/fish/meat/egg, and (6) nuts, seeds, oil, and fat. The healthy dietary behavior was categorized as “yes” or “no” in the present study.

### 2.6. Statistical Analyses

All analyses were conducted using SPSS 22.0 for Windows (SPSS Inc., Chicago, IL, USA). General characteristics were evaluated using a chi-square test, and independent sample *t*-tests were performed to compare the differences of time spent in physical activity and sedentary behavior between older adults with and without a healthy diet. For all analyses, the results are shown as means with standard deviations (M ± SD). Finally, significance was set at *p* = 0.05.

## 3. Results

Table 1 provides the results for the 127 older adults with valid data for the analysis. The mean age of those with or without a healthy diet was 69.8 ± 4.7 and 70.4 ± 5.3 years, respectively. Moreover, no differences were evident between the groups for socio-demographic factors and health conditions.

Table 2 shows subjectively and objectively measured time of intensity-specific physical activity and sedentary behavior patterns. For subjective measures, compared with those with a healthy diet, older adults without a healthy diet spent significantly less time on total leisure-time PA (60.59 ± 65.61 versus 35.09 ± 41.67, *t* = 2.09, *p* = 0.039) and leisure-time walking (39.26 ± 45.06 versus 16.04 ± 23.52, *t* = 3.75, *p* < 0.001). No differences were found for the leisure-time MVPA of the two groups (21.33 ± 37.42 versus 19.05 ± 36.74, *t* = 0.30, *p* = 0.763). Also, older adults without a healthy diet spent significantly more time than those with a healthy diet on leisure sitting time (7.47 ± 3.20 versus 8.83 ± 2.56, *t* = −2.20, *p* = 0.029). Regarding the objective measures of physical activity, the results showed that older adults with a healthy diet had a significantly higher LPA than those without a healthy diet (301.21 ± 74.94 versus 266.81 ± 89.42, *t* = 2.15, *p* = 0.033). In addition, older adults without a healthy diet had significantly higher total sedentary time (9.87 ± 1.24 versus 10.56 ± 1.21, *t* = −2.76, *p* = 0.007), sedentary bout duration (190.95 ± 73.91 versus 257.35 ± 115.24, *t* = −3.09, *p* = 0.004), sedentary bout times (4.43 ± 1.54 versus 5.66 ± 2.30, *t* = −2.85, *p* = 0.007), and sedentary break times (4.25 ± 1.54 versus 5.47 ± 2.28, *t* = −2.84, *p* = 0.007) than older adults with a healthy diet.

## 4. Discussions

This is the first study comparing the patterns of physical activity and sedentary behavior of older adults with and without a healthy diet using accelerometers and questionnaire-based measures. The main findings of this study are that older adults without a healthy diet tend to engage in more inactive and sedentary lifestyles than those with a healthy diet, regardless objective or subjective measurements. These findings may have great implications for behavioral change designers and health-promotion practitioners and indicate an urgent need to design interventions or programs to promote physical activity and reduce the sedentary behavior of older adults without a healthy diet.

An interesting finding when comparing the time spent on objectively measured physical activity between older adults with and without a healthy dietary behavior was that the time spent on total PA, MVPA, and daily step counts did not significantly differ between the two groups. However, the healthy-diet group engaged in more LPA than the unhealthy-diet group. Given that many previous studies emphasized the health benefits of engaging in LPA [23,24,25], our results may suggest that encouraging older adults without a healthy diet to accumulate more LPA could be an effective strategy to obtain optimal health benefits. In addition, when comparing the objectively measured sedentary behavior patterns of the two groups, older adults without a healthy diet engaged in higher total sedentary time, more prolonged sedentary bouts, and a longer duration of sedentary bouts than those with a healthy diet. Possibly, older adults without a healthy diet were also more likely to have a sedentary lifestyle. Since EBRBs contribute to negative impacts on older adults’ health [3,4,5], our results using objective measures may highlight the importance of decreasing the total sedentary time and 30 min sedentary bouts in older adults without a healthy diet.

Noteworthy is that when using subjective measures of leisure-time physical activity and sedentary behavior, older adults with a healthy diet engaged in significantly more leisure-time physical activity and less sedentary behavior. Questionnaire-measured physical activity and sedentary behavior were also associated with health indicators in older adults [26,27]. A possible explanation for this result is that older adults with a healthy diet may be more likely to perceive themselves as having a healthy lifestyle. This may also suggest the importance of increasing the awareness of physical activity and sedentary behavior levels among older adults without a healthy diet.

There are several limitations to this study that must be acknowledged. The cross-sectional study design cannot make strong causal inferences of the association of PA and sedentary behavior with diet quality. Possibly, if participants do not have a healthy diet, they may also have lower physical activity and spend more time on sedentary behavior. Moreover, because of a potential selection bias, the results of this study should be interpreted with caution; for instance, healthier older adults were more likely to participate in this study than unhealthy ones. Therefore, our results could not be generalized to the entire Taiwanese older population. The current study used a single question for health diet distribution and did not examine specific dietary constituents. While leisure-time physical activity and leisure sitting time are widely used and validated instruments [28,29], self-reported measurements may reflect personal biases. Finally, several potential confounders such as the basal resting metabolic rate of the participants and research duration (i.e., season’s influence) were not considered in the present study. Future studies on this issue should control for these confounders in the analyses.

In conclusion, using objective or subjective measurements, we found that older adults without a healthy diet tend to engage in more inactive and sedentary lifestyles than older adults with a healthy diet. These findings support the hypothesis of the present study and have great implications for designers of behavioral changes and health-promotion practitioners in planning tailored interventions.

## Figures and Tables

**Table 1:** Characteristics of the participants.

Variables	Healthy Diet (*n* = 94)	No Healthy Diet (*n* = 33)	*p*
Age, M ± SD	69.8 ± 4.9	70.4 ± 5.3	0.558
Gender (%)			0.291
Male	30.9%	21.2%	
Female	69.1%	78.8%	
BMI (kg/m^2^), M ± SD	24.2 ± 3.3	24.0 ± 3.6	0.752
Marital Status (%)			0.353
Married	63.8%	72.7%	
Unmarried	36.2%	27.3%	
Living status (%)			0.127
Living with others	91.5%	81.8%	
Living alone	8.5%	18.2%	
Education level (%)			0.110
University	25.5%	12.1%	
Up to high school	74.5%	87.9%	
Employment (%)			0.229
Yes	4.3%	0.0%	
No	95.7%	100.0%	
Self-rated health (%)			0.149
Good	34.8%	21.2%	
Poor	65.2%	78.8%	
Smoking habit (%)			0.872
Yes	5.3%	6.1%	
No	94.7%	93.9%	
Alcohol consumption (%)			0.071
Yes	5.3%	15.2%	
No	94.7%	84.8%	
Depression (%)			0.595
Yes	16.0%	12.1%	
No	84.0%	87.9%	
Diabetes (%)			0.153
Yes	16.0%	27.3%	
No	84.0%	72.7%	
Hypertension (%)			0.676
Yes	38.3%	42.4%	
No	61.7%	57.6%	
High blood lipid (%)			0.956
Yes	29.8%	30.3%	
No	70.2%	69.7%	

BMI: body mass index; M: mean; SD: standard deviation. The general characteristics were evaluated using a chi-square analysis.

**Table 2:** Subjectively and objectively measured physical activity and sedentary behavior patterns of older adults with and without a healthy diet.

	Variables	Healthy Diet (*n* = 94)	No Healthy Diet (*n* = 33)	*t*	*df*	*p*
		M ± SD	M ± SD			
Subjective Measurement	Total LTPA, min/week	60.59 ± 65.61	35.09 ± 41.67	2.09	125	0.039 *
	Leisure-time walking, min/week	39.26 ± 45.06	16.04 ± 23.52	3.75	106.67	<0.001 *
	Leisure-time MVPA, min/week	21.33 ± 37.42	19.05 ± 36.74	0.30	125	0.763
	Leisure sitting time, h/day	7.47 ± 3.20	8.83 ± 2.56	−2.20	125	0.029 *
Objective Measurement	Total PA, min/day	326.29 ± 77.60	289.30 ± 95.66	2.00	47.62	0.051
	LPA, min/day	301.21 ± 74.94	266.81 ± 89.42	2.15	125	0.033 *
	MVPA, min/day	25.08 ± 24.49	22.49 ± 18.91	0.55	125	0.582
	Steps, counts/day	7730.47 ± 3372.53	6674.12 ± 3371.51	1.55	125	0.124
	Total sedentary time, h/day	9.87 ± 1.24	10.56 ± 1.21	−2.76	125	0.007 *
	Sedentary bout duration, min/day	190.95 ± 73.91	257.35 ± 115.24	−3.09	41.61	0.004 *
	Sedentary bout times	4.43 ± 1.54	5.66 ± 2.30	−2.85	42.46	0.007 *
	Sedentary break times	4.25 ± 1.54	5.47 ± 2.28	−2.84	42.61	0.007 *

* *p* < 0.05; LTPA: leisure-time physical activity; PA: physical activity; LPA: Light physical activity; MVPA: Moderate-to-vigorous physical activity; M: mean; SD: standard deviation; *df*: degree of freedom. Independent sample *t*-tests were performed to compare the differences of time spent in physical activity and sedentary behavior between older adults with and without a healthy diet.
